# Differential retention and expansion of the ancestral genes associated with the paleopolyploidies in modern rosid plants, as revealed by analysis of the *extensins* super-gene family

**DOI:** 10.1186/1471-2164-15-612

**Published:** 2014-07-21

**Authors:** Lianhua Guo, Yingnan Chen, Ning Ye, Xiaogang Dai, Wanxu Yang, Tongming Yin

**Affiliations:** The Nurturing Station for the State Key Laboratory of Subtropical Silviculture, Zhejiang Agriculture and Forestry University, Lin’an, Zhejiang 311300 China; The Southern Modern Forestry Collaborative Innovation Center, Nanjing Forestry University, 159#, Longpan Road, Nanjing, 210037 China

**Keywords:** Extensin, Paleopolyploid, Purifying selection, Syntenic chromosomal block, Gene duplication manners

## Abstract

**Background:**

All modern rosids originated from a common hexapolyploid ancestor, and the genomes of some rosids have undergone one or more cycles of paleopolyploidy. After the duplication of the ancient genome, wholesale gene loss and gene subfunctionalization has occurred. Using the *extensin* super-gene family as an example, we tracked the differential retention and expansion of ancestral *extensin* genes in four modern rosids, *Arabidopsis*, *Populus*, *Vitis* and *Carica*, using several analytical methods.

**Results:**

The majority of *extensin* genes in each of the modern rosids were found to originate from different ancestral genes. In *Arabidopsis* and *Populus*, almost half of the *extensins* were paralogous duplicates within the genome of each species. By contrast, no paralogous *extensins* were detected in *Vitis* and *Carica*, which have only undergone the common *γ-*triplication event. It was noteworthy that a group of *extensins* containing the IPR006706 domain had actively duplicated in *Arabidopsis*, giving rise to a neo-*extensin* around every 3 million years. However, such *extensins* were absent from, or rare in, the other three rosids. A detailed examination revealed that this group of *extensins* had proliferated significantly in the genomes of a number of species in the *Brassicaceae*. We propose that this group of *extensins* might play important roles in the biology and in the evolution of the *Brassicaceae*. Our analyses also revealed that nearly all of the paralogous and orthologous *extensin*-pairs have been under strong purifying selection, leading to the strong conservation of the function of *extensins* duplicated from the same ancestral gene.

**Conclusions:**

Our analyses show that *extensins* originating from a common ancestor have been differentially retained and expanded among four modern rosids. Our findings suggest that, if *Arabidopsis* is used as the model plant, we can only learn a limited amount about the functions of a particular gene family. These results also provide an example of how it is essential to learn the origination of a gene when analyzing its function across different plant species.

**Electronic supplementary material:**

The online version of this article (doi:10.1186/1471-2164-15-612) contains supplementary material, which is available to authorized users.

## Background

During the evolutionary process, whole-genome duplications (WGDs) have recurred in many lineages of the angiosperms [1], leading to remarkable fluctuations in their genome sizes. Following WGDs, wholesale gene loss [1] and gene subfunctionalization [2, 3] can occur. Synteny and collinearity analyses of plant genomes have suggested that an ancient genome triplication (*γ-*triplication) event occurred in the common ancestor of *Vitis*, *Arabidopsis*, *Carica*, and *Populus*, resulting in a paleohexaploid [4]. After the *γ-*triplication event, *Arabidopsis* was affected by two recent paleopolyploidy events: β- and α- duplications. The latter was the most recent, occurring approximately 40 million years (MYs) ago [5]. In *Populus*, there was a duplication event specific to its own salicoid lineage (*P*-duplication), which occurred between the β- and α- duplication events about 65 MYs ago [6]. By contrast, in *Vitis* (grape) and *Carica* (papaya), there was only the common *γ-*triplication event and no subsequent WGDs [4, 7]. Ancient polyploidization events have affected the number of genes in various gene families. Tracking the differential retention and expansion of ancestral genes in modern plants is critical to learn the function of homologous genes across different plant species. In this study, we demonstrate the differential retention and expansion of ancestral genes in four rosids, focusing on the *extensin* super-gene family as an example.

Extensins are hydroxyproline-rich glycoproteins (HRGPs), and are members of a superfamily of plant cell-wall proteins that includes arabinogalactan proteins, extensins, and proline-rich proteins [8]. Extensins account for 1–15% of the dry weight of the cell wall of dicots [9]. In terms of their amino acid compositions, extensins are rich in hydroxyproline (Hyp), serine (Ser), and contain various amounts of tyrosine, valine, lysine, and histidine. Extensin, in a narrow sense, describes HRGPs with the characteristic Ser-Hyp-Hyp-Hyp-Hyp motif [10]. Recently, however, Showalter *et al.* [8] identified putative extensins with two or more repeats of the standard Ser-Pro-Pro-Pro and/or Ser-Pro-Pro-Pro-Pro sequences.

Extensins are fully functional after post-translational modification. The proline residues of extensins are first hydroxylated to Hyp, and then modified by arabinosyltransferases [11]. After being secreted into the cell wall, mature extensins form a network by oxidative cross-linking of several Tyr residues [12, 13]. Extensins have been implicated in nearly all aspects of plant growth and development [14]. Studies have shown that pollen- and pistil-specific extensin-like proteins play roles in reproduction [15–17]. Extensins are also involved in the responses to wounding and pathogen invasion [18, 19]. In recent years, studies on the *Arabidopsis rsh* mutant have demonstrated that extensins not only play an essential role in strengthening mature cell walls, but also in shaping the cell, positioning the cell plate during cytokinesis, and allowing normal embryo development [20]. The RSH extensin (*AtEXT*3) was thought to function exclusively in the cessation of cell growth, but recent research showed that it also has an essential role in the initiation of new cell growth [20].

Extensin genes are a special research interest in plant biology because of their biological importance. In this study, focusing on the *extensin* super-gene family as an example, we analyzed the expansion of these genes in *Arabidopsis thaliana*, *Populus trichocarpa*, *Vitis vinifera*, and *Carica papaya*, whose genomes have been affected by one or more paleopolyploidy events. We aimed to track the differential retention and expansion of the ancestral *extensins* in these four morden rosids to provide a panoramic view of the evolutionary process of a super-gene family.

## Methods

### Identification of extensins

Extensins were identified following the method described by Showalter *et al.* [8]. First, we searched for two or more SP3/SP4 repeats in protein sequences in genomic databases of *Arabidopsis thaliana* (TAIR10 release of November 2010; http://www.arabidopsis.org/), *Populus trichocarpa* (JGIv3.0, ftp://ftp.jgi-psf.org/pub/compgen/phytozome/v9.0/Ptrichocarpa/), *Vitis vinifera* (ftp://ftp.jgi-psf.org/pub/compgen/phytozome/v9.0/Vvinifera), and *Carica papaya* (ftp://ftp.jgi-psf.org/pub/compgen/phytozome/v9.0/Cpapaya/). The protein hits were subsequently scanned by InterPro (European Bioinformatics Institute) [21, 22] to find signature protein domains, including IPR006706 (extensin-2), IPR006041 (pollen Oie e 1 allergen/extensin), IPR003882 (pistil-specific extensin-like protein), IPR003883 (extensin-1), PR01217 (proline-rich extensin), and PTHR23201 (extensin, proline-rich protein).

### Identification of paralogs and orthologs

Paralogs and orthologs were identified following the method described by Blanc and Wolfe [3]. For each species, all-against-all nucleotide sequence similarity searches were performed among the transcribed sequences using BLASTN software [23]. Sequences that aligned over 300 bp and showed at least 40% identity were defined as pairs of paralogs. To identify putative orthologs between two species (A and B), each sequence from species A was searched against all sequences from species B using BLASTN. Additionally, each sequence from species B was searched against all sequences from species A. The two sequences were defined as orthologs if each of them was the best hit of the other, and if more than 300 bp of the two sequences aligned.

### Calculation of ω and Ks values

Pairwise protein sequence alignment was performed using MAFFT v6.8 [24, 25]. Then, the protein alignments were re-edited into codon-based alignments using an in-house PERL script. The codon-based alignments were converted into TREE format files using ClustalX [26] and a PAML-compatible format using DAMBE [27]. The PAML [28] -format files were further converted into NUC format. A ‘bin’ folder was created, and the data files (TREE-format file and NUC-format files) and PAML executive programs (codeml.exe, codeml.ctl) were copied into the ‘bin’. Finally, codeml.exe was run to generate the ω, dN, and dS values, where ω = dN/dS and dS = Ks.

### Phylogenetic trees construction

Protein sequences of the extensins in the four plant species were aligned using the L-INS-i software implemented in MAFFT v. 6.8 [24, 25] with the following parameters: the scoring matrix for amino acid sequences was BLOSUM62, the gap opening penalty was 2.0, and the gap extension penalty was 0.2. The derived protein alignments were re-edited into codon-based alignments using an in-house PERL script. Phylogenetic trees were reconstructed with MEGA v. 5.0 [29] using the minimum evolution (ME) and neighbour-joining (NJ) methods. The reliability of interior branches was assessed with 1,000 bootstrap re-samplings.

We constructed other phylogenetic trees using more advanced methods, including the maximum likelihood (ML) and Bayesian inference (BI) methods. The ML tree was generated with RAxML using the GTR+G model and nucleotide data sets [30]. The BI tree was generated with PhyloBayes-MPI [31] using the GTR-CAT+G4 model and nucleotide data sets. For each data set, two independent runs were executed until the maximum discrepancy between the bi-partition was less than 0.1. In both the ML and BI trees, the reliability of interior branches was assessed with 1,000 bootstrap re-samplings. On the established trees, branches supported with bootstrap values greater than 60% were joined.

### Analysis of duplication manners for extensin genes

The plant genome duplication database (PGDD; available at http://chibba.agtec.uga.edu/duplication/) is a public database to identify and catalogue plant genes in terms of intra-genome or cross-genome syntenic relationships. To identify *extensin* genes that had arisen from segmental or whole-genome duplications (S/WGD) in the genomes of the four rosid plants, we used the PGDD [32] to retrieve the syntenic chromosomal blocks (SCBs) associated with the expansion of *extensin* genes through S/WGDs. First, in the PGDD, we detected all of the chromosomal blocks that contained *extensin* genes in each of the four rosids. Then, we retrieved their syntenic blocks within and between species from the PGDD. The *extensin* genes duplicated through S/WGDs were identified based on gene collinearity on SCBs. In this analysis, the counterparts of a particular *extensin* gene on an SCB may have been retained as *extensins*, subfunctionalized into non-*extensins*, or completely lost. Additionally, some of the syntenic blocks could have been completely lost after the ancient S/WGDs.

*Extensin* genes expanded through tandem duplication (TD) were inferred following the method described by Tuskan *et al.* [6], with a Smith-Waterman alignment E value of ≤ 10^-25^ and a 100-kb window. If the paralogous *extensins* had expanded neither through W/SGD nor TD, they were considered to have proliferated via other duplication strategies [33].

### Reconstructing a tentative phylogeny of the large paralogous group in *Arabidopsis*

We used a hierarchical clustering method to reconstruct a tentative phylogeny of the large paralogous group in *Arabidopsis*, as follows [3]: (1) all sequences in the group were treated as separate clusters; (2) the Ks values for all possible pairs of clusters were compared; (3) the pair of clusters with the smallest Ks value was replaced by a single new cluster containing all of their sequences; (4) the median Ks value was chosen to represent the duplication event that gave rise to the two merged clusters; and (5) steps 2–4 were repeated until all sequences were contained in a single cluster. When two clusters (A and B) contained more than one sequence, their associated Ks value in step 2 was the median Ks obtained for all possible pairs of any sequence from A and B. The gene neutral evolutionary rate in *Arabidopsis* was reported to be 1.5 × 10^-8^ [34]. The duplication times for the paralogous *extensin* pairs were estimated by Ks/(2 × 1.5 × 10^-8^) according to Blanc and Wolfe’s calculation [3].

## Results

### *Extensin*genes in four modern rosids

The four studied rosids, *A. thaliana*, *P. trichocarpa*, *C. papaya*, and *V. vinifera*, originate from a common paleohexapolyploid ancestor. Based on the WGDs in each species, the multiplicity ratio for an ancestral gene in the above species should be 4:2:1:1. We retrieved 46, 37, 18, and five putative *extensin* genes from the genomes of *A. thaliana*, *P. trichocarpa*, *C. papaya*, and *V. vinifera*, respectively (Additional file 1). In *V. vinifera*, 94.5% of its genome demonstrates the occurrence of the paleohexaploidy, and this species appears to have the closest karyotype to that of the common ancestor [7]. When *V. vinifera* was taken as the baseline, the current ratio of *extensin* genes among the four genes was 9.2: 7.4: 3.6: 1. Compared with *V. vinifera*, the other three species showed higher-than-expected current ratios of *extensins*. Therefore, *V. vinifera* may have lost *extensin* genes faster than did the other species, or *extensin*s may have expanded faster in the other three species than in *V. vinifera*.

A detailed examination (Additional file 2) revealed that the most common type of extensins were those containing the PR01217 (PRICHEXTENSN) domain. This group made up the largest proportion of extensins in each of the four modern rosids, accounting for 54%, 89%, 95%, and 80% of all of the extensins in *Arabidopsis*, *Populus*, C*arica* and *Vitis*, respectively. *Arabidopsis* has 18 extensins containing the IPR006706 (Extensin-2) domain; this group has specifically and remarkably proliferated only in *Arabidopsis* (accounting for 39.1% of all extensins in *Arabidopsis*). This group of extensins was rare or absent from the other three rosids.

### *Extensin*paralogs and orthologs

The paralogous and orthologous *extensins* within and between species are listed in Table 1. In *Arabidopsis*, five paralogous groups containing 22 *extensins* were identified. In *Populus*, there were seven paralogous groups containing 18 *extensins*. Thus, nearly half of the *extensins* were associated with intra-specific duplication in *Arabidopsis* and *Populus*. By contrast, there were no paralogous duplicates in *Carica* and *Vitis*, which have undergone only the common *γ* triplication event and no subsequent WGDs. There was a large paralogous group containing 12 *extensins* in *Arabidopsis* (Table 1), all encoding proteins with the IPR006706 (Extensin-2) domain.

**Table 1:** **Paralogous and orthologous** ***extensins*** **identified in four modern rosid plants**

	***Arabidopsis thaliana***	***Populus trichocarpa***	***Carica papaya***	***Vitis vinifera***
Paralogous extensins within species	*A2*-*A6*-*A7*-*A8*-*A9*-*A10*-*A11*-*A12*-*A13*-*A15*-*A16*-*A17*; *A18-A19-A20*; *A24-A28-A49*; *A25-A31*; *A26-A27*	*P1-P2*; *P5-P7-P6*; *P10-P12*; *P18- P19*; *P20-P22-P28*; *P26-P17*; *P34-P35-P36-P37*	—	—
Orthologous extensins between species	*P5*-*A35*; *P6*-*A39*; *P29*-*A44*; *C18*-*P30*; *C6-P6*; *C7-P24*; *C8-P20*; *V1-C6*; *V1-P5*			

The observed ratio of *extensins* (37:18) was close to the expected ratio (2:1) based on the paleopolyploidy of *Populus* and *Carica*. These two species shared the most orthologous pairs (Table 1), followed by *Populus* and *Arabidopsis*. Although *Carica* was the closest relative of *Arabidopsis* among the four species (both belong to the Brassicales), they had no orthologous *extensins*. There were also no orthologous *extensins* between *Vitis* and *Arabidopsis*. One pair of orthologous extensins was detected between *Carica* and *Vitis*.

The ratio of non-synonymous substitutions per non-synonymous site (dN) to the synonymous substitutions per synonymous site (dS) is an indicator of natural selection [35]. We can evaluate the driving force shaping the evolution of *extensins* based on the value of ω = dN/dS. If ω < 1, the gene is undergoing purifying selection, resulting in gene function becoming more convergent. When ω > 1, gene function will be more divergent. Our calculations (Additional file 3) clearly showed that all the paralogous and orthologous *extensins* within and between species were under strong purifying selection, with an overall mean value of ω = 0.15. Purifying selection was not evident for only one paralogous pair, *P22*-*P28*, in *Populus* (ω = 0.9765). The average ω for paralogous pairs was 0.24 in poplar, and 0.12 in *Arabidopsis*. Therefore, paralogous pairs of *extensins* have been under stronger purifying selection in *Arabidopsis* than in poplar.

### Phylogenetic analysis of *extensin*genes in four modern rosids

**Figure 1:**
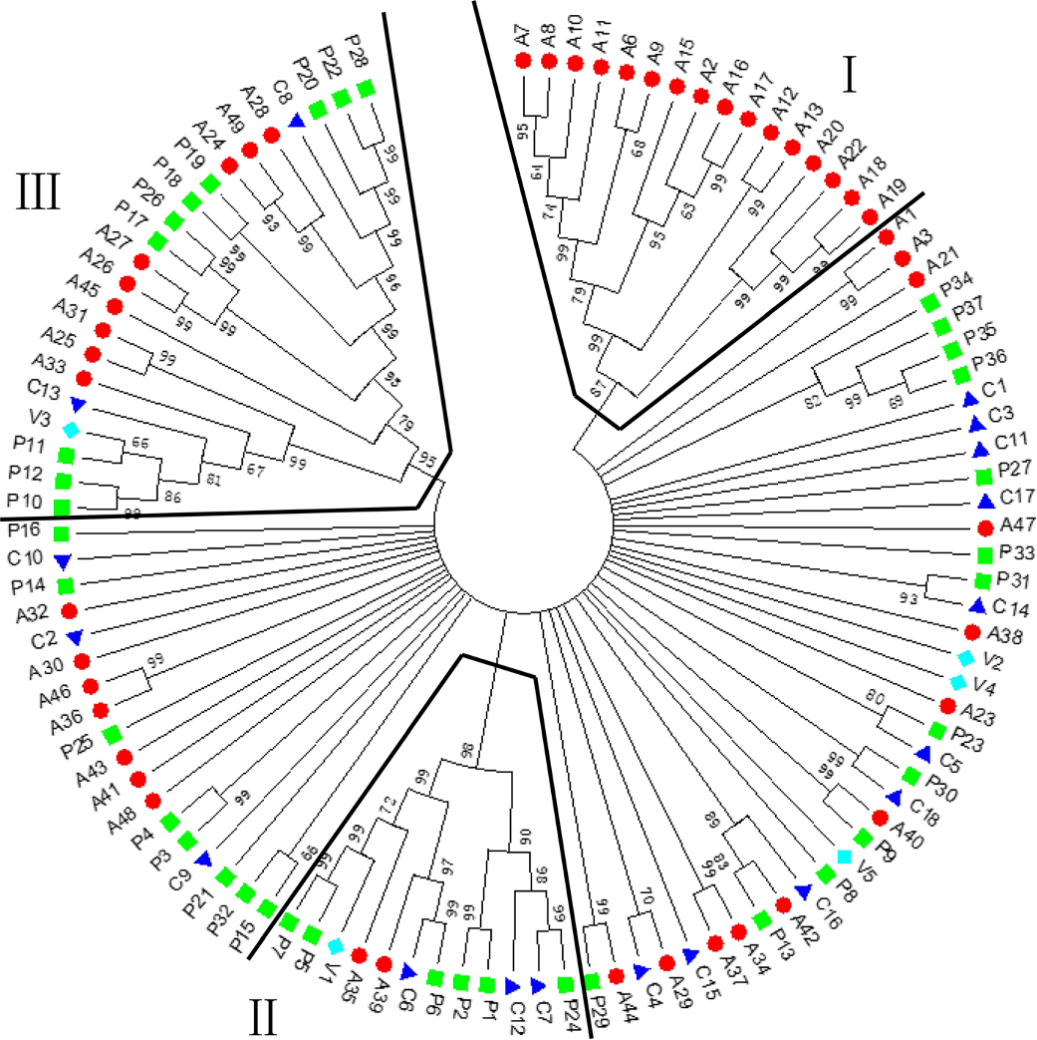
**Phylogenetic tree of** ***extensins*** **constructed by the ME method with MEGA for the four modern rosids.**

Phylogenetic trees were constructed with MEGA [29] using the ME method (Figure 1) and the NJ method (Additional file 4). The ME and NJ trees showed identical topologies. A total of 106 genes were distributed among 43 branches with bootstrap values ≥60%, and formed three distinct clades (Figure 1). Clade I consisted of 16 *extensins* from *Arabidopsis*. This clade consisted of two sub-clades, one containing *A20*, *A22*, *A18* and *A19*, and the other containing the large paralogous group of 12 *Arabidopsis extensins* described above. The fact that all of the *extensins* in this clade were from *Arabidopsis*, combined with the results of the paralog analysis (Table 1), indicated that all of the *extensins* in clade I were intra-specific duplicates in *Arabidopsis*. Clade II consisted of 12 *extensins*; two from *Arabidopsis*, six from *Populus*, three from *Carica*, and one from *Vitis. Extensins* in this clade included two paralogous groups from *Populus* (*P1*-*P2* and *P5*-*P6-P*7) and six orthologous pairs shared by the four modern rosids (*C7-P24*, *P5-A35*, *P6-A39*, *C6-P6*, *V1-P5* and *V1-C6*). Therefore, most of the *extensins* in this clade represented the ancestral relics shared between species. The *extensins* in this clade had expanded most dramatically in *Populus*. Clade III contained 22 *extensins*: nine from *Arabidopsis*, 10 from *Populus*, two from *Carica*, and one from *Vitis*. Paralogous groups in this clade were *A24-A28-A49*, *A26-A27*, and *A25-A31* from *Arabidopsis*, and *P20-P22-P28*, *P18-P19*, *P17-P26*, and *P10-P12* from *Populus*. Clade III contained only one orthologous pair, *C8-P20*. Therefore, most of the *extensins* in this clade were intra-specific duplicates that have expanded in *Arabidopsis* and *Populus*. As well as the three distinct clades described above, there was a small clade containing four paralogous *extensins* from *Populus* (*P34*, *P35*, *P36*, and *P37*). These represented *extensins* that have specifically expanded within the *Populus* genome.

Next, we compared the phylogenetic trees described above with those generated using more advanced ML and BI methods. The ML tree constructed using the GTR+G model is shown in Additional file 5. The BI tree constructed using the GTR-CAT+G4 model is shown in Additional file 6. The topology of the ML tree was roughly consistent with that of the BI tree for branches with greater than 80% bootstrap support. When the ML and BI trees were compared with the ME and NJ trees at ≥60% bootstrapping support, the topology of the formers pair of trees differed significantly from that of the latter pair. Clades II and III, which were distinct in the ME and NJ trees, were collapsed in the ML and BI trees. However, when we separately combined the results for branches with more than 80% bootstrap support in the ME and NJ trees and did the same in the ML and BI trees, the combined results of the former were almost completely consistent with the combined results of the latter. The only exception was the emergence of *A1* and *A3*, which was only supported with a bootstrap value ≥80% in the ME and NJ trees.

We further evaluated the phylogenetic relationship of paralogous and orthologous *extensins* in the four rosids (Table 1). We found that any of the paralogous and orthologous pairs were located in the same clade on the ME and NJ trees, suggesting the ME and NJ trees were ideal to infer the phylogenetic relationship of the paralogous and orthologous *extensins* with bootstrap support ≥60%.

### Expansion manners of *extensin*genes

**Figure 2:**
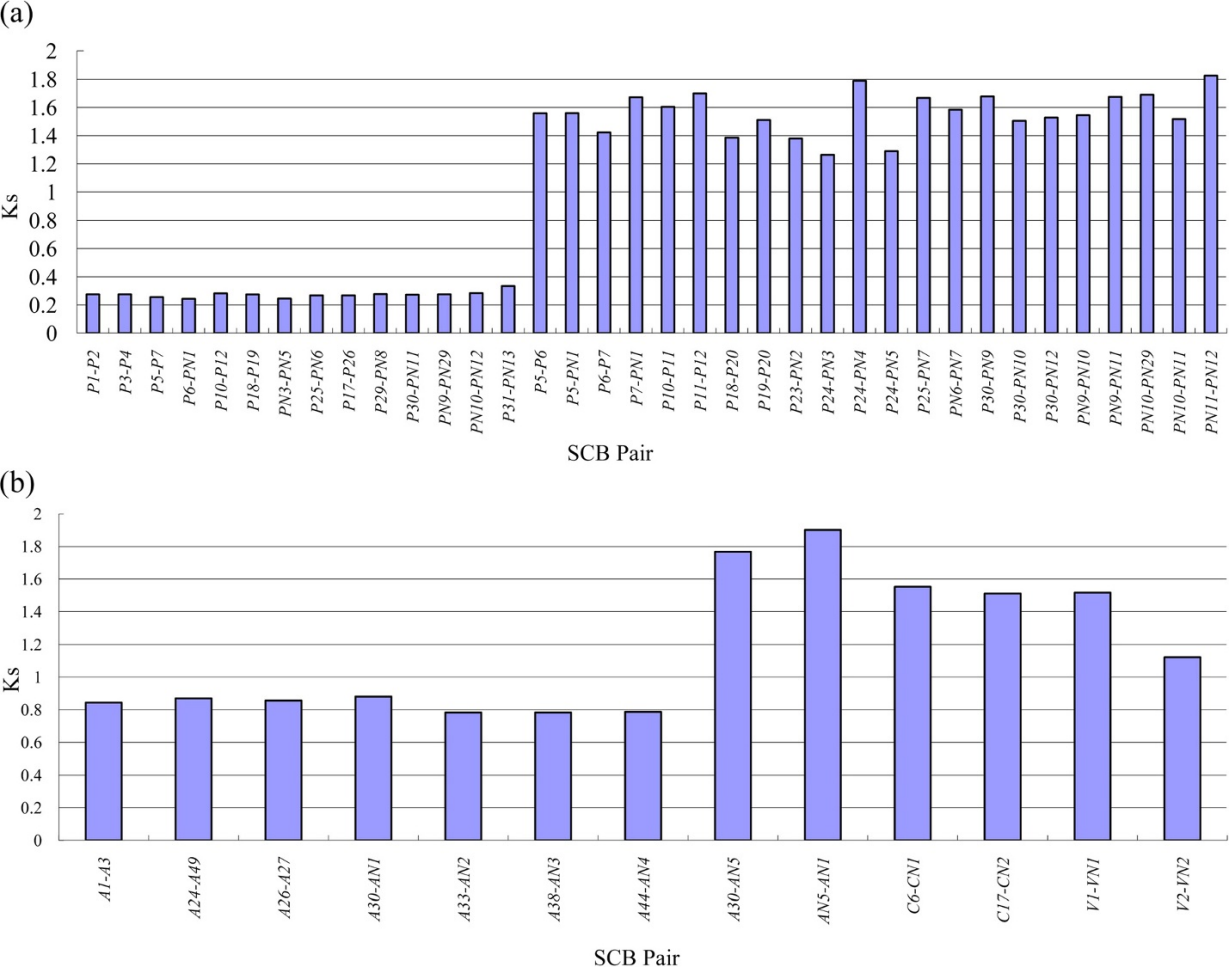
**Median Ks values of SCB pairs associated with the expansion of** ***extensins*** **within the genomes of each species. a**: Within the genome of *Populus*. **b**: Within the genomes of the other three species.

From the PGDD [32], we first retrieved the SCBs associated with the expansion of *extensins* within the genome of each species (Additional file 7). The number of *extensins* that arose from S/WGD varied among the four rosids. We detected 10, 21, two and two *extensin* genes associated with S/WGDs in *Arabidopsis*, *Populus*, *Carica*, and *Vitis*, respectively. Because the duplicated genes located on a SCB pair have duplicated simultaneously, the median Ks value of duplicated genes in SCBs can be used to infer the associated WGDs [5]. In *Populus*, the overall median Ks value of the duplicated genes related to the γ triplication event was 1.54, and that associated with the *P*-WGD was 0.27, as reported by Tang *et al.* [36]. We detected 36 SCB pairs associated with the expansion of *extensin* genes within the genome of *Populus* (Additional file 7). The median Ks of duplicated genes in different SCBs showed two distinct ranges (Figure 2a): 0.2437–0.3345, and 1.2633–1.7896. According to the calculations of Tang *et al.* [36], we proposed that SCBs with median Ks values in the range of 0.2437–0.3345 were associated with the *P*-WGD, and those with median Ks values in the range of 1.2633–1.7896 were likely related to the most ancient γ-triplication event (Figure 2a). For example, SCBs containing *P5* and *P7* likely arose from the *P*-WGD, as did the SCBs containing *P6* and *PN1*. However, the median Ks value of any SCB combination (*P5-PN1*, *P5-P6*, *P7-PN1*, *P6-P7*) across the above two SCB pairs was in the range of 1.2633–1.7896. This finding suggested that the archetypal chromosomal blocks of these two SCB pairs (*P5-P7*, *P6-PN1*) were duplicates resulting from the γ-triplication event. The counterparts of *extensins* on many SCBs were found to have subfunctionalized into non-*extensins* (indicated by an “N” between the first letter and the number in the code name).

We further examined the SCBs associated with the expansion of *extensins* within the genomes of the other three species. In *Arabidopsis*, the median Ks values associated with β- and γ-WGDs were close to the saturation median Ks value of 2.00 [36]. Thus, the β- and γ-paleopolyploidies were indistinguishable based on the Ks value. The overall median Ks value associated with α-duplication was reported to be 0.86 [36]. In total, 10 *extensin* genes were associated with α-WGD in *Arabidopsis*, and the median Ks value of SCBs containing these genes were in the range of 0.783–0.881 (Figure 2b). Among these SCBs, the SCB pair *A30-AN1* had a median Ks value of 0.881, suggesting this pair resulted from α-WGD. The SCB pair *A30-AN5* had a median Ks value of 1.7678, suggesting that this pair was associated with β- or γ-paleopolyploidy. In both cases, the counterparts of *extensins* on the corresponding SCBs had subfunctionalized into non-*extensins* (*AN1*, *AN5*). In *Carica* and *Vitis*, the overall median Ks value of SCBs associated with γ triplication was 1.76 and 1.22, respectively [36]. In this study, we detected two SCBs containing *extensin* genes (*C6-CN1*, *C17-CN2*) in *Carica*; these pairs had median Ks values of 1.5526 and 1.5113, respectively (Figure 2b). This finding suggested that both duplicates resulted from γ-WGD. In *Vitis*, the SCB pair *V1-VN1* had a median Ks value of 1.5164 (Figure 2b). The two chromosomal blocks of this SCB pair were located on *Vitis* chromosomes 1 and 14, and each had 37 duplicated genes in identical order. The other SCB pair, *V2-VN2*, had a median Ks value of 1.1221. The formation of the above two SCB pairs was associated with γ-WGD in *Vitis*.

In the above analyses, we analyzed the SCBs associated with the expansion of *extensin* genes within the genome of each species. Using the PGDD database [32], we also examined the SCBs associated with the expansion of *extensins* between species (Additional file 8). When a gene-collinearity analysis was conducted between species, we detected more *extensins* resulting from S/WGDs. For example, when the gene-collinearity analysis was conducted within a single species, we found that *A33* and *AN2* in *Arabidopsis* originated from α-WGD. When the gene-collinearity analysis was conducted between species, we found that the chromosomal blocks containing *A33*, *AN2*, *A25*, and *A31* were all orthologous SCBs of the chromosomal block containing *C13* in *Carica*. Therefore, these four genes originated from a common ancestral gene, and the paleopolyploidy associated with *A25* and *A31* should be more ancient than the α-WGD event. However, whether *A25* and *A31* resulted from β- or γ-WGD could not be determined because the median Ks values between β- and γ-SCBs in *Arabidopsis* were close to saturation. In *Arabidopsis*, *extensins* arising from α-WGD can be identified with certainty, but those resulting from β- and γ-WGD are undistinguishable. Similarly, the results of the gene-collinearity analysis between species revealed that *A35* and *A39* were associated with β- or γ-WGD in *Arabidopsis*.

**Figure 3:**
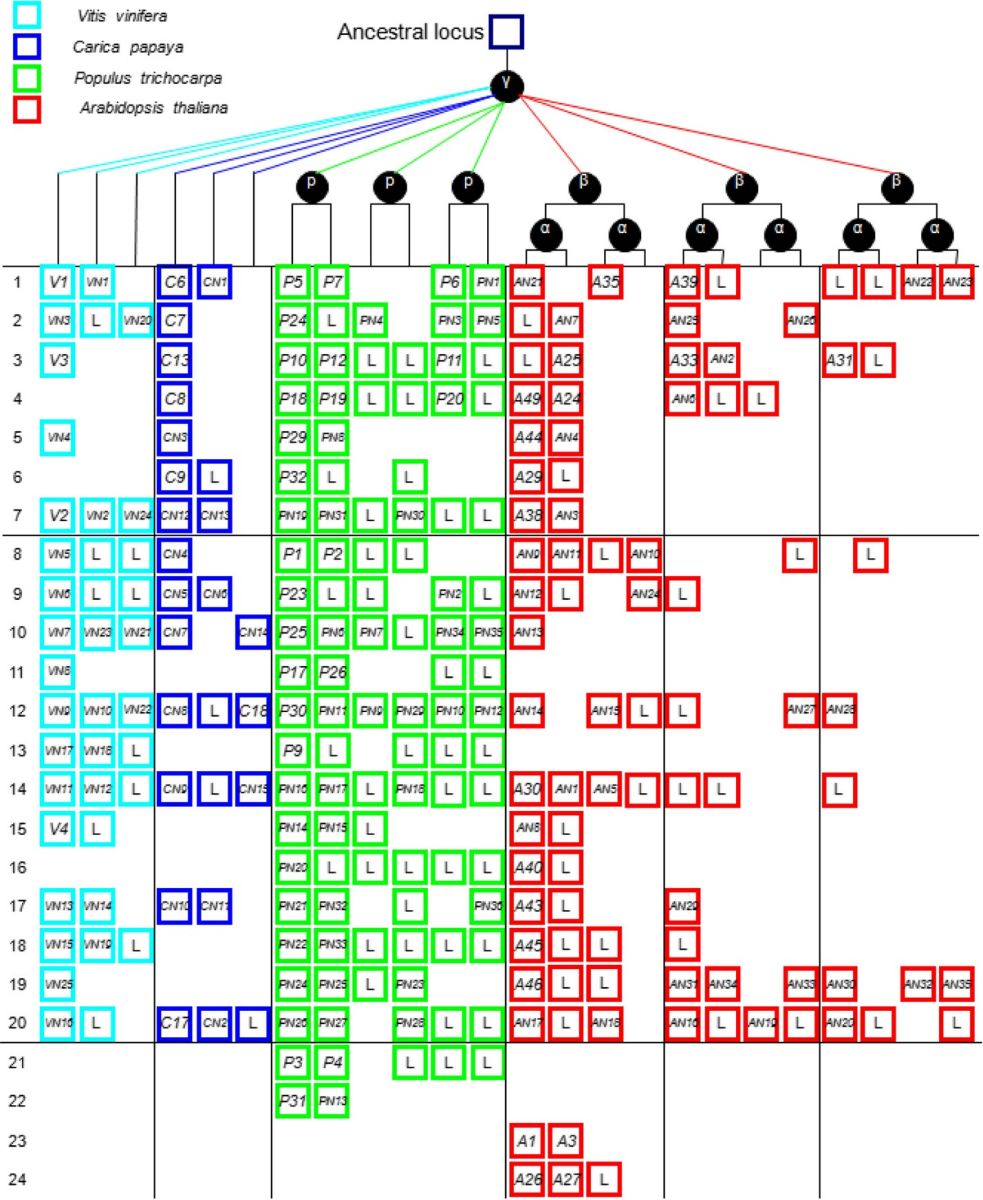
**Panoramic picture to visualize the differential retention and expansion of the ancestral** ***extensins*** **associated with paleopolyploidy events that have occurred in four modern rosids.** Notes: Square represents a SCB duplicated through paleopolyploidy events within and between species. Codes in the square correspond to associated *extensin* genes. Genes in the same line are thought to have originated from the same ancestral gene. Genes coded with a letter followed by a number (e.g., *V1*) represent genes retained as *extensins*; genes coded with an “N” between the letter and the number (e.g., *CN1*) represent those that have subfunctionalized into non-*extensins*; “L” represents duplicated *extensin* that has been lost, but the corresponding SCB has been retained; blank positions correspond to situations where the whole SCBs has been completely lost.

Based on the gene-collinearity analysis within and between species, we established a panoramic picture of the differential retention and expansion of the ancestral *extensins* associated with paleopolyploidy in the four modern rosids (Figure 3). The retention and expansion of 24 ancestral *extensins* in these four modern rosids could be tracked unambiguously through gene collinearity analyses. The duplicates of these ancestral genes through S/WGDs were differentially retained in each species. We detected 19, 23, 6, and 4 *extensin* genes associated with paleopolyploidy in *Arabidopsis*, *Populus*, *Carica*, and *Vitis*, respectively (Figure 3). All of the other duplicates of these 24 ancestral genes that arose through S/WGDs were either subfunctionalized into non-*extensins* or completely lost (Figure 3). For instance, all genes shown in the first line of Figure 3 originated from the same ancestral gene (ancestral *extensin*-1). The genes that originated from this ancestral gene through ancient S/WGDs, including *V1*, *C6*, *P5*, *P7*, *P6*, *A35* and *A39*, were remained as *extensins*; whereas the duplicates of this ancestral *extensin*, including *VN1*, *CN1*, *PN1*, *AN21*, *AN22*, and *AN23* were subfunctionalized into non-*extensins*. Three duplicates of this ancestral *extensin* were lost from *Arabidopsis*, but the corresponding SCBs were retained (represented by an “L” at the corresponding position). Eight whole-SCBs have been completely lost from these four modern rosids (blank at the corresponding positions).

Besides *extensins* associated with paleopolyploidy, eight *extensins* in *Arabidopsis* were found to result from TDs, including *A2*, *A17*, *A7*, *A8*, *A9*, and *A10* in the large paralogous group and another paralogous pair, *A18-A19* (Table 1). In *Populus*, four *extensins* in the paralogous group (*P34*, *P35*, *P36*, and *P37*; Table 1) were generated through TDs; whereas in *Carica* and *Vitis*, no *extensin* genes had expanded through TD. Among the *extensins* expanded through TDs, none originated from the 24 ancestral *extensins* shown in Figure 3.

Regardless of the type of gene duplication event, all paralogous genes were likely duplicated from the same ancestral gene [37]. In *Arabidopsis*, we detected eight *extensins* (*A*6, *A11*, *A1*2, *A13, A15*, *A16*, *A20*, and *A28*) that had expanded through duplication manners other than S/WGD or TD. In *Populus*, two paralogous *extensins*, *P22* and *P2*8, were identified to have duplicated via other manners. In *Carica* and *Vitis*, no paralogous *extensins* expanded via duplication manners other than S/WGD or TD. Among the *extensins that* expanded through other duplication manners, *A28* (paralogs of *A24* and *A49*, Table 1), *P22* and *P2*8 (paralog of *P20*, Table 1) originated from the same ancestral gene, one of the 24 ancestral *extensins* mentioned above (line 4, Figure 3).

The ortholog analysis revealed that *C18* and *P30* were an orthologous pair. A detailed examination revealed that *C18* is located on a small contig in the *C. papaya* genome assembly [27]; this region contains only two genes and did not qualify for the gene collinearity analysis. *C18* and *P30* originated from the same ancestral gene (line 12, Figure 3).

Besides the *extensins* that expanded via traceable events, there were 11 other *extensins* unique to *Arabidopsis* (*A21*, *A2*2, *A23*, *A32*, *A34*, *A36*, *A3*7, *A41*, *A42*, *A47*, *A48*), eight unique to *Populus* (*P8*, *P13*, *P14*, *P15*, *P16*, *P21*, *P27, P33*), 11 unique to *Carica* (*C1*, *C2*, *C3*, *C*4, *C5*, *C10*, *C11*, *C1*2, *C14*, *C1*5, *C16*) and one unique to *Vitis* (*V5*). We propose that these *extensins* might represent the oldest relics of ancient *extensins* differentially retained in each species. Alternatively, some might be ancient intra-specific duplicates, but their paralog-ship was no longer traceable because of severe gene divergence or erosion of duplication signatures.

### Large paralogous *extensin*group in Arabidopsis

**Figure 4:**
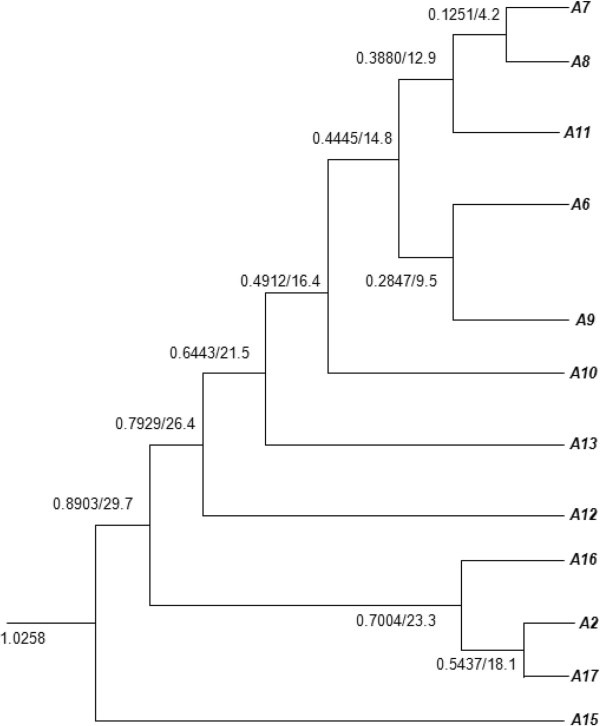
**Tentative phylogeny constructed for a large paralogous group of** ***extensins*** **in** ***Arabidopsis*** **.**

We detected a large paralogous group containing 12 *extensins* genes (*A2*, *A6*, *A7*, *A8*, *A9*, *A10*, *A11*, *A12*, *A13*, *A15*, *A16*, and *A17*) in *Arabidopsis* (Table 1). A paralog analysis suggested that all of these genes originated from a common ancestral gene. The ancestral *extensin* was retained and specifically expanded in the genome of *Arabidopsis*, but was lost from the genomes of the other three rosids. The remarkable and specific proliferation of this ancestral *extensin* in *Arabidopsis* suggests that this group of extensin genes plays an unusually important role in the biology of *Arabidopsis*. Using the method of Blanc and Wolfe [3], we reconstructed a tentative phylogeny of this large paralogous group (Figure 4). In *Arabidopsis*, the Ks values of paralogous pairs associated with the most recent α-WGD range of 0.72–0.99 [5].

According to the Ks values shown in Figure 4, *A15* is the most ancient *extensin* in this paralogous group, diverging from the other 11 *extensins* before the α-WGD. Around 29.7 MYs ago, *A16*, *A2* and *A17* further diverged from the remaining eight *extensins*. As shown in Figure 3, none of these *extensins* expanded through S/WGDs. Therefore, apart from *A15*, all of the other *extensins* in this paralogous group duplicated after the α-WGD. Among them, *A2*, *A7*, *A8*, *A9*, *A10*, and *A17* expanded through TDs, while the others proliferated through other duplication manners. The genes in this paralogous group duplicated very actively. On average, duplication occurred around every 3 MYs, and the most recent duplication event occurred approximately 4 MYs ago between *A7* and *A8* through TD.

## Discussion

*A. thaliana*, *P. trichocarpa*, *C. papaya*, and *V. vinifera* originate from a common paleohexaploid ancestor, and more recent paleopolyploidy events have recurred in the genomes of *Populus* and *Arabidopsis*. Paleopolyploidy events lead to gene duplication, which is believed to play a major role in evolutionary innovation [38]. Ancient genome duplications offer opportunities for the evolution of new genes [39] or genes with modified functions [40], changes in gene dosages, and the formation of new gene arrangements [36].

Relationships among genes in modern plants can be inferred based on sequence similarity, phylogenetic distance, and syntenic SCBs. However, each method has its own constraints. For instance, sequence similarity may have severely eroded during the evolutionary process for many of the ancient duplicates. Consequently, the paralog-ship for such duplicates would no longer be traceable based on sequence similarity. For example, our analyses showed that *A1* and *A3* are duplicates resulting from α-WGD in *Arabidopsi*s, and *P3* and *P4* are duplicates that arose from *P*-WGD in *Populus* (Figure 3), but these paralogous pairs were not detected in the paralog analysis based on gene similarity (Table 1). More ancient duplicates could be tracked based on gene collinearity on SCBs within and between species. Yet, based on the gene collinearity analysis alone, we could not detect the paralogous pairs that arose via duplication manners other than S/WGDs (e.g., the large paralogous group unique to *Arabidopsi*s; Table 1).

It is problematic to infer evolutionary relationships for homologous genes among different lineages based on phylogenetic distance alone. Gene variation rates vary among lineages. Therefore, inferring evolutionary relationships based on phylogenetic trees can produce incongruous results, because the drastic differences in rates may lead to incorrect trees that are artifacts because of long-branch attractions [41]. Therefore, to reconstruct the evolutionary relationships for members of a gene family across plant species, it is essential to combine all of the above analytical methods.

Based on gene collinearity on SCBs within and between species, we built a panoramic picture to display the differential retention and expansion of 24 ancestral *extensins* among the four modern rosids (Figure 3). Our analyses showed that seven (line 1–7) of these ancestral *extensins* were retained in more than two species, but only two (line 1 and line 3) were retained in all four modern rosids. By contrast, 17 of the ancestral *extensins* were retained in only one of the four rosids. Thus, most of the *extensins* in each modern rosid are descendants of different ancestral genes. This finding suggests that, using *Arabidopsis* as the model plant, we can only learn a limited amount about the functions of a gene family. If *Arabidopsis* is the only study material, we might not resolve the function of genes uniquely retained in the other rosids (e.g., *V4* in *Vitis*, *C17* in *Carica*, and *P31* in *Populus*). Similarly, genes uniquely retained in *Arabidopsis* may have a specific and indispensable function in the species. For example, studies on *A1* (*Extensin*-1) and *A3* (*Extensin*-3) have demonstrated their importance in maintaining normal cell wall function in *Arabidopsis* [12, 14, 20, 42]. In particular, the RSH extensin (Extensin-3) was shown to play an essential role in the initiation of new cell growth [20]. Because *A1* and *A3* were uniquely retained in *Arabidopsis*, it remains unknown whether their function was compensated for by other extensins in the other three rosids, or whether such function was totally lost.

Considering the paleopolyploidy events that occurred in each species, there should be three ancestral loci in *Carica* and *Vitis*, because they only underwent the γ-triplication event. There should be six ancestral loci in *Populus*, because it underwent both γ-triplication and *P*-duplication, and 12 in *Arabidopsis* because it underwent γ-triplication, and then β- and α-duplications. However, such extreme values were not observed for any of the ancestral *extensins*. After the paleopolyploidy events, the exponential growth in gene numbers is often tempered by massive and progressive gene death in the subsequent diploidization process [4]. In this study, about 91.3% *extensins* associated with paleopolyploidies were found to have subfunctionalized into non-*extensins* or to have been completely lost from these four modern rosids (Figure 3). The convergent restoration of some genes to singleton status after multiple rounds of duplication in independent lineages suggests that there may be selective advantages for the organism to have only a single copy of these genes [43]. In most cases, we found that only one copy of the duplicated *extensins* resulting from ancient WGDs had been retained (Figure 3). In another study, such *extensins* were identified as “duplication resistant”; that is, only one copy per nucleus was adaptive [36].

As well as the *γ-*triplication event, *Arabidopsis* was affected by two more recent paleopolyploidy events (β- and α-duplication). In *Populus*, there was only one additional duplication (*P*-duplication), and the *P*-duplication in *Populus* was more ancient than the most recent α-duplication in *Arabidopsis* [5, 6]. However, *Arabidopsis* retained fewer *extensins* that proliferated through S/WGDs than did *Populus.* This may result from rapid substitutions in *Arabidopsis*. The *γ-*triplication event apparently occurred in the common ancestor of the four modern rosids. However, the median Ks between *γ-*paleologs in *Arabidopsis* (close to the saturation value of 2.00) was higher than that in *Populus* (1.54), *Carica* (1.76), and *Vitis* (1.22) [36]. This result suggests that more rapid substitutions occurred at synonymous sites in *Arabidopsis* than in the other three rosids. The high median Ks between *γ-* paleologs in *Arabidopsis* may be related to the more extensive chromosome rearrangements that occurred in *Arabidopsis*. There were three fusions, two translocations, and one inversion event during the 5 MYs after *Arabidopsis lyrata* diverged from *A. thaliana* [34]. This would give a rate estimate of 0.6 rearrangement/MY in the genome of these two species. The more extensive rearrangements of the chromosomal segments that occurred in *Arabidopsis* would destroy collinearity [44]. In species that have undergone several ancient WGDs, the more recent WGDs tend to obscure the collinearity from the more ancient ones [36]. Additionally, more SCBs were completely lost (blank sites in Figure 3) from *Arabidopsis* than from *Populus*. This result indicates that heavier contraction of chromosome blocks in *Arabidopsis* than in *Populus* accompanied the WGDs in the past. This idea is consistent with the fact that these two lineages originated from a common ancestor, and that *Arabidopsis* contains more paleopolyploidies, yet has a much smaller genome than that of *Populus*.

Besides the *extensins* expanded through S/WGDs, some *extensins* proliferated more actively through duplication manners other than S/WGD. *Extensins* in the latter category were more abundant in *Arabidopsis* and *Populus* than in *Carica* and *Vitis*. Among the genomes of the four rosids, that of *Vitis* shows the closest karyotype to that of their common ancestor [7]. Our analyses showed that almost all *Vitis extensins* are old relics of ancestral *extensins* from the common paleohexaploid ancestor (Figure 3). Both *Carica* and *Vitis* were only affected by the γ-WGD event, but *Carica* contains many more *extensins* than does *Vitis*. Among the *Carica extensins*, only seven show clear evidence of being relics of the common ancestor (Figure 3). Although we cannot exclude the possibility that some of the remaining *extensins* might represent ancient intra-specific duplicates that are no longer traceable, it is certain that there are no recently duplicated *extensins* in *Carica*. The fact that there are more *extensins* in *Carica* than in *Vitis* may imply that it uses more of these proteins in the development process and for adaptation to the environment.

A striking finding of this study is that *extensins* with the IPR006706 motif specifically expanded in *A. thaliana*. Such *extensins* were rare or completely absent from the other three rosids (Additional file 1). Both *C. papaya* and *A. thaliana* are in the *Brassicaceae*, yet *C. papaya* has only one *extensin* with the IPR006706 motif (*C11*). Selection after a duplication event contributes substantially to gene novelty, and hence, to functional divergence of genes in plants [45]. The ω values for paralogous *extensins* encoding proteins with the IPR006706 domain indicate that this group has been subjected to strong purifying selection. This finding highlights the importance of the function of this highly conserved gene group in *Arabidopsis*.

Referring to the phylogenetic tree at the phytozome website (http://www.phytozome.net/alyrata.php), we further analyzed the expansion of such *extensins* in close relatives of *A. thaliana*, including *A. lyrata*, *Capsella rubella*, and *Brassica rapa*. All of these species are members of the *Brassicaceae*. Similar to the case in *A. thaliana*, such *extensins* had also significantly expanded in these lineages. We detected 10, 7, and 15 *extensins* with an IPR006706 motif in *A. lyrata*, *C. rubella*, and *B. rapa*, respectively. Like in *Arabidopsis*, more than 50% of these *extensins* had expanded through TDs in each species. Additionally, nine, six, three, and six of these *extensins* were shared orthologs between species in *A. thaliana*, *A. lyrata*, *C. rubella* and *B. rapa*, respectively. Thus, the expansion of this *extensins* group occurred after the progenitor of the above four lineages diverged from *Carica*.

## Conclusions

Based on sequence similarity, phylogenetic distance and gene collinearity on the SCBs, we tracked the differential retention and expansion of ancestral extensin genes among four modern rosids. The results revealed that most of the *extensins* in each species are descendants of different ancestral genes. We also detected a group of *extensins* that specifically expanded in the *Arabidopsis* genome. An important group of *extensins* has been retained only in *Arabidopsis*. Whether their function was compensated for by other extensins or such function was totally lost in the other rosids remains unknown. These findings highlight that we can only learn a limited amount about the functions of a particular gene family using *Arabidopsis* as the model plant. This study also highlights the importance of learning the origin of a gene when analyzing its function across different plant species.

## Availability of supporting data

The data set(s) supporting the results of this article hyperlink to dataset(s) in http://www.arabidopsis.org (TAIR10 release of November 2010), ftp://ftp.jgi-psf.org/pub/compgen/phytozome/v9.0/Ptrichocarpa/ (*Populus trichocarpa*), ftp://ftp.jgi-psf.org/pub/compgen/phytozome/v9.0/Vvinifera (*Vitis vinifera*), and ftp://ftp.jgi-psf.org/pub/compgen/phytozome/v9.0/Cpapaya/ (*Carica papaya*).

The relevant primary data have been deposited in TreeBase, with Study Accession URL: http://purl.org/phylo/treebase/phylows/study/TB2:S16026.

## Authors’ information

Lianhua Guo: Ph.D Nanjing Forestry University. He is now a faculty member at Zhejiang Agriculture & Forestry University.

Yingnan Chen: Ph.D, faculty member of Nanjing Forestry University.

Ning Ye: Ph. D, faculty member of Nanjing Forestry University.

Xiaogang Dai: Ph.D, Nanjing Forestry University.

Wanxu Yang: Graduate student at Nanjing Forestry University.

Tongming Yin: Ph.D, laboratory director and full time professor at Nanjing Forestry University.

## Electronic supplementary material

Additional file 1: ***Extensins*** **identified in four modern rosids.** (XLS 38 KB)

Additional file 2: **Number of extensins with different signature protein domains in four modern rosids.** (XLS 30 KB)

Additional file 3: **ω values for the paralogous and orthologous pairs of** ***extensins.*** (XLS 34 KB)

Additional file 4: **Phylogenetic tree of** ***extensins*** **constructed by NJ method with MEGA for four modern rosids.** (JPEG 719 KB)

Additional file 5: **Phylogenetic tree of** ***extensins*** **constructed by ML method with GTR+G model.** (JPEG 312 KB)

Additional file 6: **Phylogenetic tree of** ***extensins*** **constructed by BI method with GTR-CAT+G4 model.** (JPEG 278 KB)

Additional file 7: ***Extensins*** **in syntenic chromosomal blocks identified within each species.** (XLSX 39 KB)

Additional file 8: ***Extensins*** **in syntenic chromosomal blocks identified between species.** (XLS 49 KB)

## Abbreviations

PGDD: The plant genome duplication database
S/WGD: Segmental or whole-genome duplications
SCBs: Syntenic chromosomal blocks
TD: Tandem duplication.
