# Supplementary figures and images for: Differential retention and expansion of the ancestral genes associated with the paleopolyploidies in modern rosid plants, as revealed by analysis of the extensins super-gene family
Source: BMC Genomics. 2014 Jul 21;15(1):612. doi: 10.1186/1471-2164-15-612 (PMC4117974; doi:10.1186/1471-2164-15-612)

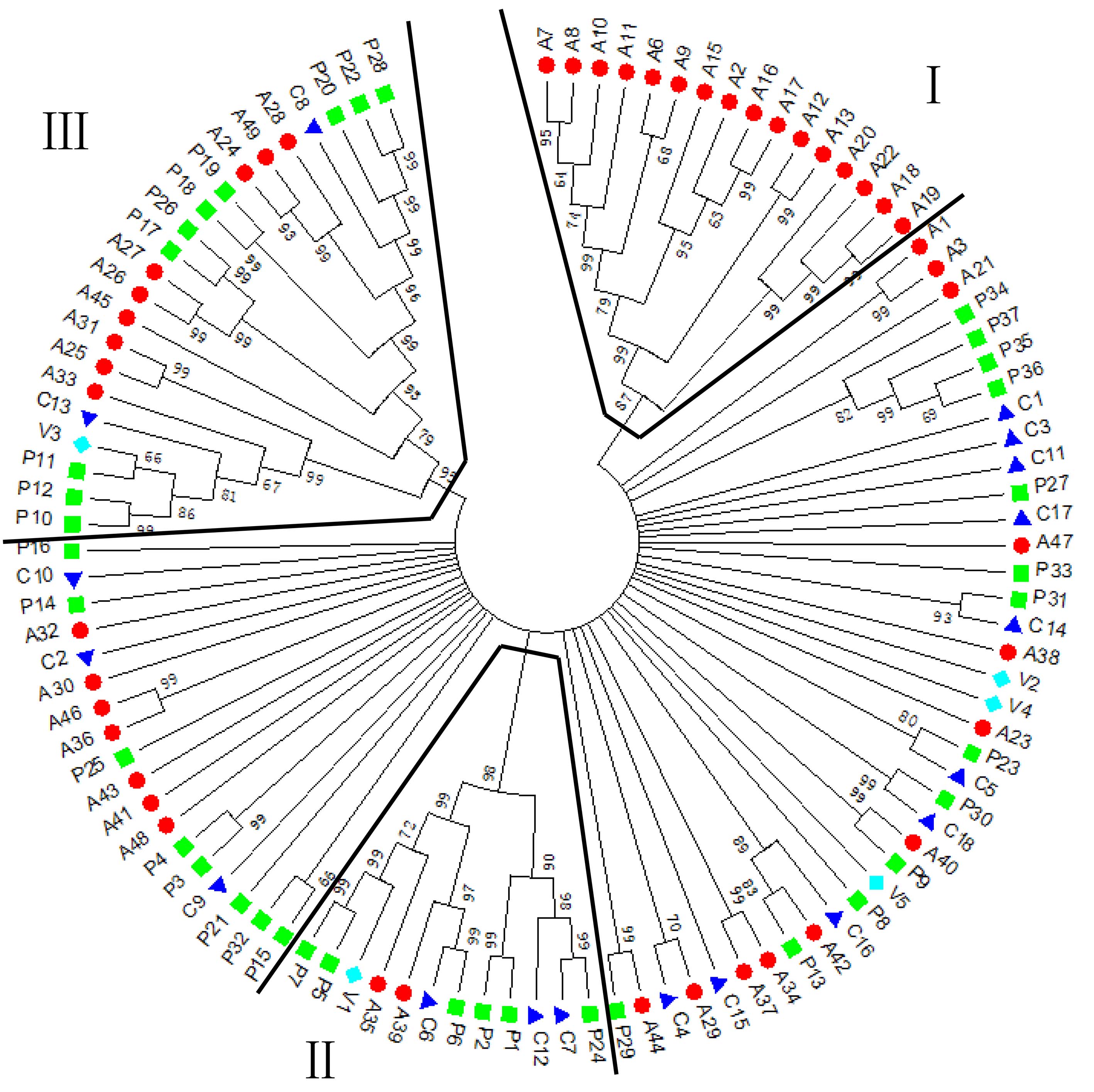

Supplement: Supplementary file 4 — Additional file 4: Phylogenetic tree of extensins constructed by NJ method with MEGA for four modern rosids. (JPEG 719 KB) [file 12864_2014_6305_MOESM4_ESM.jpeg]

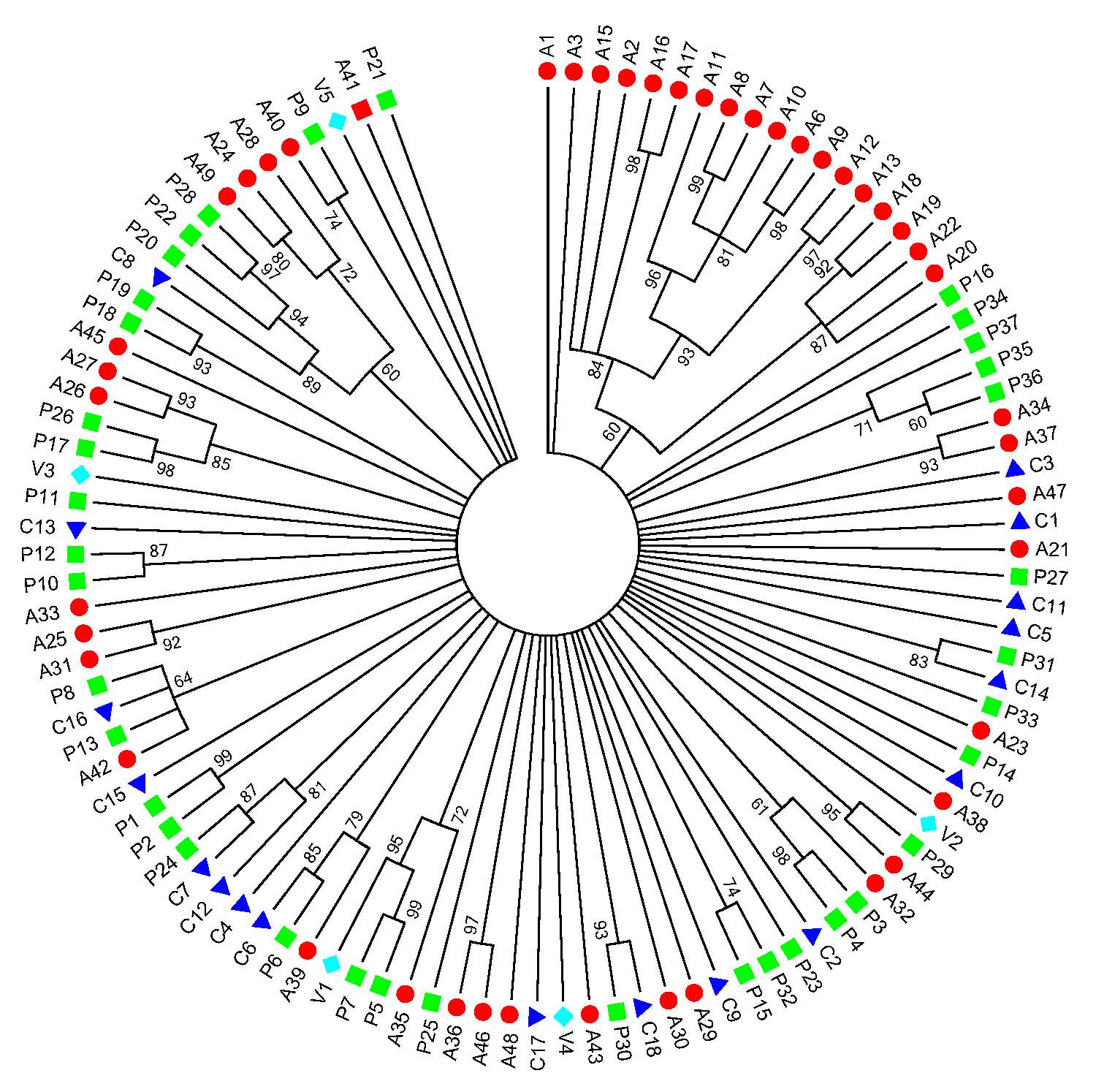

Supplement: Supplementary file 5 — Additional file 5: Phylogenetic tree of extensins constructed by ML method with GTR+G model. (JPEG 312 KB) [file 12864_2014_6305_MOESM5_ESM.jpeg]

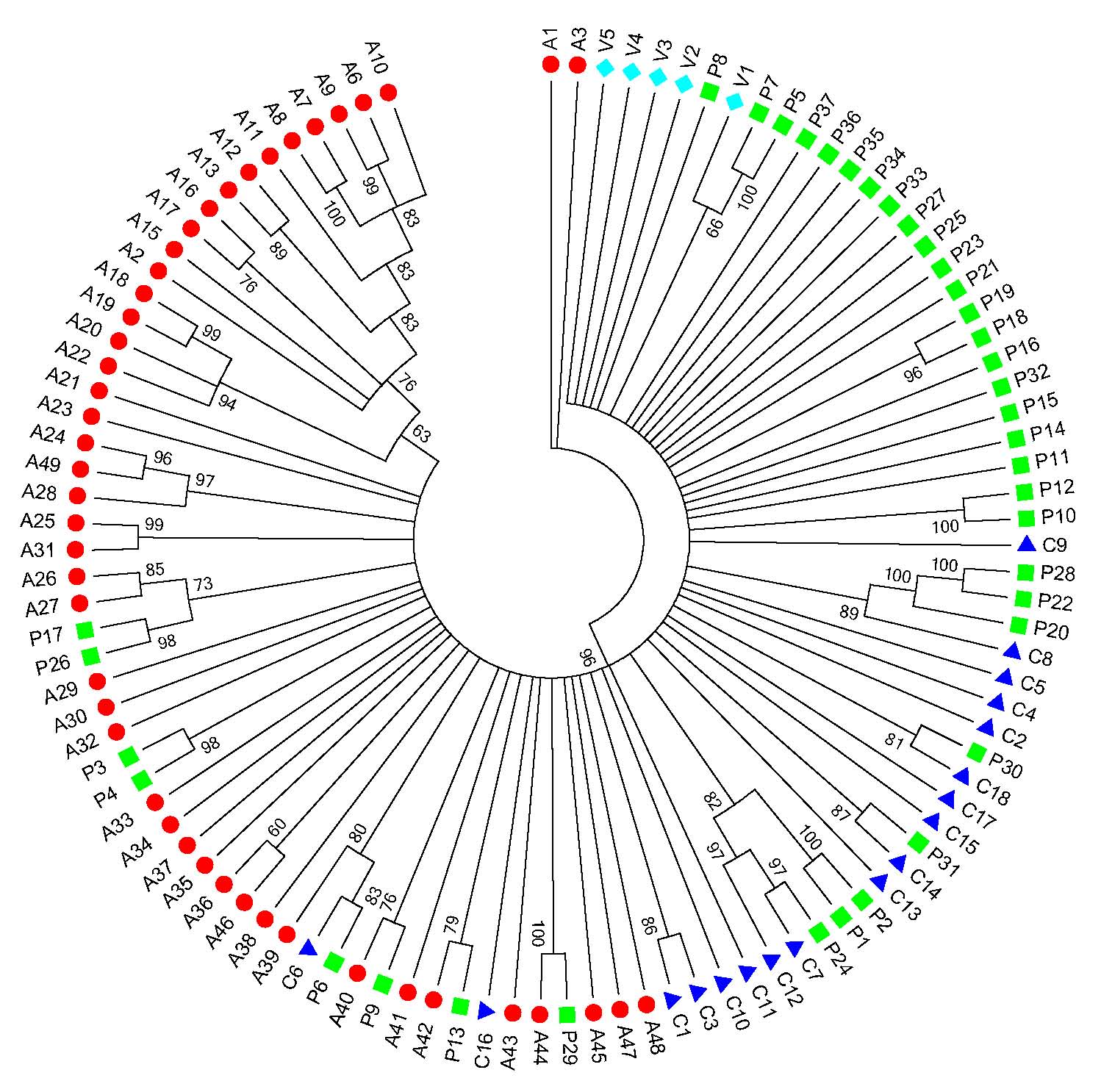

Supplement: Supplementary file 6 — Additional file 6: Phylogenetic tree of extensins constructed by BI method with GTR-CAT+G4 model. (JPEG 278 KB) [file 12864_2014_6305_MOESM6_ESM.jpeg]
